# Model for fitting longitudinal traits subject to threshold response applied to genetic evaluation for heat tolerance

**DOI:** 10.1186/1297-9686-41-10

**Published:** 2009-01-14

**Authors:** Juan Pablo Sánchez, Romdhane Rekaya, Ignacy Misztal

**Affiliations:** 1 Departamento de Producción Animal, Facultad de Veterinaria, Universidad de León, Campus de Vegazana, León, 24071, Spain; 2 Animal and Dairy Science Department, University of Georgia, 425 River Road, Athens, GA, 30602, USA

## Abstract

A semi-parametric non-linear longitudinal hierarchical model is presented. The model assumes that individual variation exists both in the degree of the linear change of performance (slope) beyond a particular threshold of the independent variable scale and in the magnitude of the threshold itself; these individual variations are attributed to genetic and environmental components. During implementation via a Bayesian MCMC approach, threshold levels were sampled using a Metropolis step because their fully conditional posterior distributions do not have a closed form. The model was tested by simulation following designs similar to previous studies on genetics of heat stress. Posterior means of parameters of interest, under all simulation scenarios, were close to their true values with the latter always being included in the uncertain regions, indicating an absence of bias. The proposed models provide flexible tools for studying genotype by environmental interaction as well as for fitting other longitudinal traits subject to abrupt changes in the performance at particular points on the independent variable scale.

## Introduction

Reaction norm models have been proposed as an alternative for fitting Genotype by Environment interactions (GxE) in evolutionary biology and animal breeding [1]. In reaction norm models, the environment is often described by a continuous variable, and the phenotypes are partially explained by the regression of the genotypic values on the environmental values. When an environmental variable is observed on a continuous scale (*i.e*., temperature), it is expected to have a direct one-to-one relationship between the environmental scale and values. Consequently, the reaction norm model can be fitted by regressing the genotypic values on the observed environmental scale [2,3]. When the observed environmental scale is not continuous (*i.e*., herd classes), the genotypic values can be regressed on the effect of the categorical variable defining the different environments using, for example, least squared means of the class effects [4] or inferring the environmental values jointly with the remaining set of parameters in the model [5].

In animal breeding applications of reaction norm models, it was assumed that both the mean and the variances are either continuous, monotone functions of the environmental values [4,6] or that they are such only when the environmental values exceed a certain threshold [2,7,3]. In past studies involving thresholds, the same threshold was assumed for all animals, and it was estimated based on the quality of the fit of the average performances as a function of environmental values.

The objective of this study was to present a Bayesian hierarchical model for fitting a longitudinal trait showing an abrupt linear change at some value of the independent variable. Simulations were inspired by reaction norm models, and the procedure postulates that the effect of the environmental variable is not existent until it exceeds a certain unknown value particular for each individual with data. Furthermore, the model allows for partitioning individual variability on the threshold into genetic and environmental components.

## Methods

### Model and Prior specification

A general description of hierarchical Bayesian modelling can be found in [8]. Here the first stage of the hierarchy describes the data generating process, or the conditional distribution of the observed phenotypes given the model parameters. The following model was assumed:

*y*_*ijk *_= *CG*_*k *_+ *μ*_*j *_+ *ϕ*_*j *_× max{0, *THI*_*ij *_- *τ*_0, *j*_} + *ε*_*ijk*_,

where *y*_*ijk *_is the *i*^*th *^observation measured on animal j in contemporary group k (*CG*_*k*_), and *THI*_*ij *_is the temperature and humidity index [2,7] associated with the i^th ^observation of animal j. Random variables *μ*_*j*_, *ϕ*_*j *_and *τ*_0, *j *_associated with the animal j represent an intercept (*μ*_*j*_), or individual value in the absence of heat stress, slope (*ϕ*_*j*_), or a change in the performance per unit of change in the THI index above the individual threshold (*τ*_0, *j*_). In this study, the heat load function [7] was defined in a way that was similar to previous studies on genetics of instantaneous heat stress on daily milk production [2]. Finally, *ε*_*ijk *_is a random homoskedastic error term associated with each particular observation.

The data was assumed to be normally distributed as follows:

$$y_{ijk}|CG_{k},\mu_{j},\varphi_{j},\tau_{0,j},THI_{ij},\sigma_{\varepsilon }^{2}\sim N\left(CG_{k}+\mu_{j}+\varphi_{j}\times \max\left\{0,THI_{ij}-\tau_{0,j}\right\},\sigma_{\varepsilon }^{2}\right).$$

The second stage of the hierarchy consisted of specifying prior distributions for all parameters in the first stage.

$$\sigma_{\varepsilon }^{2}\sim U\left(0,+\infty \right)$$

$$CG\sim \prod_{k=1}^{K}U\left(-\infty ,+\infty \right)$$

where U indicates the uniform distribution and K is the number of levels of the contemporary group effect.

The underlying variables associated with the j^th ^animal, *μ*_*j*_, *ϕ*_*j *_and *τ*_0, *j*_, were assumed to follow the multivariate normal distribution:

(1) $$\left(\mu ,\varphi ,\tau_{0}|\beta_{\mu },\beta_{\varphi },\beta_{\tau_{0}},a_{\mu },a_{\varphi },a_{\tau_{0}},R_{0}\right)\sim MVN\left(X\beta +Za,I⊗R_{0}\right),$$

where $\beta^{\prime }=\left({\beta^{\prime }}_{\mu },{\beta^{\prime }}_{\varphi },{\beta^{\prime }}_{\tau_{0}}\right)$, $a^{\prime }=\left({a^{\prime }}_{\mu },{a^{\prime }}_{\varphi },{a^{\prime }}_{\tau_{0}}\right)$, and *μ*, *ϕ*_0 _and *τ*_0 _are vectors including scalar parameters of individuals (*μ*_*j*_, *ϕ*_*j *_and *τ*_0, *j*_).

Parameters of a given individual were considered to be conditionally independent and affected at their mean level by systematic (***β***_*μ*_, ***β***_*ϕ *_and $\beta_{\tau_{0}}$) and genetic effects (**a**_*μ*_, **a**_*ϕ *_and $a_{\tau_{0}}$); the residual (co)variance matrix between underlying variables was **R**_0_, which is equivalent to a (co)variance matrix between permanent environmental effects on the observed measures scale.

In a third hierarchical stage, prior distributions for systematic and genetic effects and the residual (co)variance matrix between underlying variables were defined. Systematic effects were considered to be uniformly distributed, and genetic effects were assumed to follow a multivariate normal distribution according to the genetic infinitesimal model [9]:

$$\left(a_{\mu },a_{\varphi },a_{\tau_{0}}|G_{0}\right)\sim MVN\left(0,A⊗G_{0}\right),$$

where **G**_0 _is the (co)variance matrix between the additive genetic effects for the underlying variables. The residual (co)variance matrix was assumed to follow a uniform distribution.

In the fourth and last hierarchical stage, a prior distribution was assigned to the genetic (co)variance matrix for the underlying variables. A uniform distribution was assumed as in the case of the residual (co)variance matrix.

### Fully conditional posterior distributions

The fully conditional posterior distributions must be obtained in order to perform a Bayesian MCMC estimation procedure using the Gibbs sampler algorithm. After defining the joint posterior distribution as the product of the conditional likelihood and all the prior distributions [8], the terms involving the parameter of interest in the joint posterior distribution were retained. For the model described, all the fully conditional posterior distributions are exactly the same as those described for a hierarchical model assuming intercept and linear terms [10], except those involving the individual thresholds. For all the position parameters, both in the first and second hierarchical stages, the fully conditional posterior densities were proportional to normal distributions; the fully conditional distribution for the residual variance in the first stage followed a scaled inverted chi squared distribution, and the genetic and residual (co)variance matrices in the third and second stages followed inverted Wishard distributions.

For the thresholds, the fully conditional posterior distribution had the following form:

$$\begin{array}{lll} p\left(\tau_{0,j}|y,CG,\mu_{j},\varphi_{j},\sigma_{\varepsilon }^{2},\beta ,a_{\mu ,j},a_{\varphi ,j},a_{\tau_{0},j},R_{0}\right) & \propto & p\left(y|\tau_{0,j},CG,\mu_{j},\varphi_{j},\sigma_{\varepsilon }^{2}\right)\times \\ & & p\left(\tau_{0,j}|\mu_{j},\varphi_{j},\beta ,a_{\mu ,j},a_{\varphi ,j},a_{\tau_{0},j},R_{0}\right), \end{array}$$

which can be explicitly expressed as:

$$\begin{array}{l} p\left(\tau_{0,j}|y,CG,\mu_{j},\varphi_{j},\sigma_{\varepsilon }^{2},\beta ,a_{\mu ,j},a_{\varphi ,j},a_{\tau_{0},j},R_{0}\right)\propto \\ \exp\left\{\sum_{i\in J}-{\frac{\left(y_{ijk}-CG_{k}-\mu_{j}-\varphi_{j}\times \max\left\{0,THI_{ij}-\tau_{0,j}\right\}\right)}{2\sigma_{\varepsilon }^{2}}}^{2}\right\}\times \\ \exp\left\{-\frac{{\left(\tau_{0,j}-\left(\left(X_{ij}\beta_{\tau_{0}}+a_{\tau_{0},j}\right)-\frac{\left(\left(\mu_{j}-X_{ij}\beta_{\mu }-a_{\mu ,j}\right)r^{\mu ,\tau_{0}}+\left(\varphi_{j}-X_{ij}\beta_{\varphi }-a_{\varphi ,j}\right)r^{\varphi ,\tau_{0}}\right)}{r^{\tau_{0},\tau_{0}}}\right)\right)}^{2}}{\frac{2}{r^{\tau_{0},\tau_{0}}}}\right\}, \end{array}$$

The first term comes from the likelihood; J refers to the subset of records belonging to animal j. The second term comes from the prior (second hierarchical stage); note that the relationship between the animal j and the other individuals in the population are taken into account throughout the given values of the additive genetic effects. In this second factor, scalars r^i, j ^refer to the relevant elements of the inverse of **R**_0_, which is the residual (co)variance matrix in the second hierarchical stage. This fully conditional posterior distribution does not have a known closed form; thus a Metropolis step [11] was used to sample from it.

In the model presented, the definitions of the genetic and phenotypic variances in a given environment are slightly more difficult than in the standard reaction norm models because a non-linear function of random correlated variables is involved. Thus, a Monte Carlo approximation of the phenotypic variance was determined for a particular value of THI during the measurement day. For example, in a particular environment (THI value) this quantity was calculated in the r^th ^round of the Gibbs sampler:

$${\hat{\sigma }}_{P}^{2\left[r\right]}=\frac{{\left({\hat{p}}^{\left[r\right]}-E\left({\hat{p}}^{\left[r\right]}\right)\right)}^{\prime }\left({\hat{p}}^{\left[r\right]}-E\left({\hat{p}}^{\left[r\right]}\right)\right)}{n-1}+{\hat{\sigma }}_{\varepsilon }^{2\left[r\right]}$$

where n is the number of records, and ${\hat{p}}^{\left[r\right]}$, with expected value $E\left({\hat{p}}^{\left[r\right]}\right)$, is a vector of size n with typical elements defined as below:

$${\hat{p}}_{ij}^{\left[r\right]}=\left({\hat{a}}_{\mu ,j}^{\left[r\right]}+{\tilde{e}}_{\mu ,j}\right)+\left({\hat{\bar{\varphi }}}^{\left[r\right]}+{\hat{a}}_{\varphi ,j}^{\left[r\right]}+{\tilde{e}}_{\varphi ,j}\right)\times \max\left\{0,THI_{h}-\left({\hat{\bar{\tau }}}_{0}^{\left[r\right]}+{\hat{a}}_{\tau_{0},i}^{\left[r\right]}+{\tilde{e}}_{\tau_{0},j}\right)\right\}.$$

In this expression ${\hat{a}}_{\mu ,j}^{\left[r\right]},{\hat{a}}_{\varphi ,j}^{\left[r\right]}$ and ${\hat{a}}_{\tau_{0},j}^{\left[r\right]}$ are the sampled values for the additive genetic effects for the animal j during the r^th ^iteration; ${\tilde{e}}_{\mu ,i},{\tilde{e}}_{\varphi ,i}$ and ${\tilde{e}}_{\tau_{0},i}$ are random deviates sampled from $MVN\left(0,{\hat{R}}_{0}^{\left[r\right]}\right)$, where ${\hat{R}}_{0}^{\left[r\right]}$ is the value of the residual (co)variance matrix in the second hierarchical stage sampled; ${\hat{\bar{\tau }}}_{0}^{\left[r\right]}$ and ${\hat{\bar{\varphi }}}^{\left[r\right]}$ are sampled values of the overall mean for the threshold level and slope. They were computed during the r^th ^iteration by applying the appropriate vectors of linear contrast to the sampled vector of systematic effects, ${\hat{\beta }}_{\tau_{0}}^{\left[r\right]}$ and ${\hat{\beta }}_{\varphi }^{\left[r\right]}$. Finally, in the equation of the overall phenotypic variance, ${\hat{\sigma }}_{\varepsilon }^{2\left[r\right]}$ is the value of the residual variance in the first hierarchical stage. We used the aggregated phenotypes (*i.e*.${\hat{\bar{\varphi }}}^{\left[r\right]}+{\hat{a}}_{\varphi ,j}^{\left[r\right]}+{\tilde{e}}_{\varphi ,j}$) instead of the sampled values $\mu_{j}^{\left[r\right]},\varphi_{j}^{\left[r\right]}$, and $\tau_{0,j}^{\left[r\right]}$ to avoid the variation due to systematic effects in the second hierarchical stage.

For the case of the additive variance, its Monte Carlo approximation can be computed by calculating this quantity in each round of the Gibbs Sampler:

$${\hat{\sigma }}_{a}^{2\left[r\right]}=\frac{{\left({\hat{u}}^{\left[r\right]}-E\left({\hat{u}}^{\left[r\right]}\right)\right)}^{\prime }A^{-1}\left({\hat{u}}^{\left[r\right]}-E\left({\hat{u}}^{\left[r\right]}\right)\right)}{N-1}$$

where N is the number of animals in the pedigree; **A**^-1 ^is the inverse of the additive relationship matrix; ${\hat{u}}^{\left[r\right]}$ is a vector of overall additive genetic effects sampled during the iteration r; and $E\left({\hat{u}}^{\left[r\right]}\right)$ is the expected value of the random variable ${\hat{u}}^{r}$. The j^th ^element of the vector ${\hat{u}}^{\left[r\right]}$ was computed in each round of the Gibbs sampler using this expression:

$${\hat{u}}_{j}^{\left[r\right]}={\hat{a}}_{\mu ,j}^{\left[r\right]}+\left({\hat{\bar{\varphi }}}^{\left[r\right]}+{\hat{a}}_{\varphi ,j}^{\left[r\right]}\right)\times \max\left\{0,THI_{h}-\left({\hat{\bar{\tau }}}_{0}^{\left[r\right]}+{\hat{a}}_{\tau_{0},j}^{\left[r\right]}\right)\right\}$$

where ${\hat{\bar{\tau }}}_{0}^{\left[r\right]},{\hat{\bar{\varphi }}}^{\left[r\right]},{\hat{a}}_{\mu ,i}^{\left[r\right]},{\hat{a}}_{\varphi ,i}^{\left[r\right]}$ and ${\hat{a}}_{\tau_{0},i}^{\left[r\right]}$ have the same meaning as those previously described in the equation for ${\hat{p}}_{ij}^{\left[r\right]}$. Note that non-zero expected values are considered in the equations for computing both phenotypic and genetic variances; the derived random variables, ${\hat{u}}^{\left[r\right]}$ and ${\hat{p}}^{\left[r\right]}$, are non-linear functions of random correlated variables, thus their expected values are non-zero [12]. Also note that the relationships between records were not considered when computing the phenotypic variance due to complexity.

Based on these computed variance components, relevant genetic parameters and other genetic quantities can be easily defined for different environments (THI values). For example, heritability or expected genetic response to a selection index could be defined for different environmental values [13].

### Data

Simulated data sets were used to investigate the performance of the Bayesian implementation of the model described above.

Different combinations of heritabilities and correlations for the underlying variables were investigated: low (0.1), medium (0.2) and high (0.5) heritabilities; and low (0.2, 0.3) and high (0.7, 0.9) correlations, in absolute value. In addition, two different data set designs were considered, approximately 20 (S20) and 10 (S10) records per animal. Thus, 12 different scenarios were investigated, and for each one ten replications were run.

For both data size scenarios the same genetic structure was considered but with different sizes. For S20 in the first generation, 40 males and 200 females were generated, and in the second generation, each sire was mated to five females, producing four full sibs from each mating. Thus, the entire population consisted of 1,040 animals. For S10 in the first generation, 80 males and 400 females were generated, and in the second generation, each sire was again mated to five females, producing four full sibs. In this case the entire population consisted of 2080 animals. This genetic structure resembles prolific species populations like swine or rabbit.

For both data structures 21,500 records were generated according to the described model and assigned to the total number of animals in the population. For generating records only an overall mean (with a value of 90) was considered in the first hierarchical stage as the CG effect, and overall means for the threshold (19) and for the slope (-0.5) were the only considered systematic effects in the second hierarchical stage. THI values were generated by sampling from a Normal distribution with mean 18.0 and variance 10.0, resembling the distribution of THI values in a temperate climate.

### Gibbs Sampler implementation

For each replication, a Gibbs Sampler algorithm was run for 100,000 rounds, of which the first 10,000 were discarded as burn-in period; afterwards one tenth of the rounds were retained. The threshold level was sampled via a Metropolis step by using a proposal density that was normally distributed and centered on the previous value of the threshold. The variance of the proposal density was constant across animals. During the burn-in period, the value of the variance of the proposal was tuned for an average acceptance rate of around 0.5 under all the scenarios. In a post-Gibbs analysis, the convergence of the chains were assessed both by visual inspection of the trace plots for the most relevant parameters and through the Geweke test [14], in addition the effective sample size (ESS) was computed using the function effective Size () from the coda package in R [15].

## Results

Tables 1 and 2 show the results of the simulation averaged over 10 replications for the 12 investigated scenarios. For all the parameters and models, the true values were well within the uncertain regions, which is an empirical indication of the unbiasedness of the inferential method. In addition the means for all the parameters were very close to their respective true values.

**Table 1:** Parameter estimates for 6 parameter scenarios when 20 records were considered per animal (averages over 10 replications)

	**1**				**2**				**3**			
	True	PM^a^	PSD^b^	ESS^c^	True	PM	PSD	ESS	True	PM	PSD	ESS

***μ*_T_**	**19**	18.96	0.15	352	**19**	19.10	0.16	416	**19**	19.08	0.16	399
**h^2^_I_**	**0.5**	0.52	0.06	2110	**0.2**	0.20	0.05	583	**0.1**	0.14	0.05	318
**h^2^_S_**	**0.5**	0.56	0.08	617	**0.2**	0.23	0.07	392	**0.1**	0.12	0.06	112
**h^2^_T_**	**0.5**	0.48	0.18	91	**0.2**	0.36	0.15	98	**0.1**	0.37	0.16	95
***ρ*_g, I-S_**	**0.3**	0.26	0.11	818	**0.3**	0.30	0.23	301	**0.3**	0.54	0.27	75
***ρ*_g, I-T_**	**-0.2**	-0.21	0.24	159	**-0.2**	-0.23	0.36	63	**-0.2**	-0.06	0.36	61
***ρ*_g, S-T_**	**-0.2**	-0.31	0.23	141	**-0.2**	-0.19	0.33	99	**-0.2**	0.02	0.39	83
***ρ*_p, I-S_**	**0.3**	0.35	0.09	768	**0.3**	0.31	0.06	601	**0.3**	0.30	0.05	507
***ρ*_p, I-T_**	**-0.2**	-0.23	0.23	129	**-0.2**	-0.22	0.16	209	**-0.2**	-0.29	0.14	217
***ρ*_p, S-T_**	**-0.2**	-0.15	0.23	109	**-0.2**	-0.21	0.14	214	**-0.2**	-0.25	0.12	203
***σ* ^2^_e_**	**10**	9.97	0.10	8142	**10**	9.98	0.10	9000	**10**	9.99	0.10	9000

	**4**				**5**				**6**			

	True	PM	PSD	ESS	True	PM	PSD	ESS	True	PM	PSD	ESS

***μ*_T_**	**19**	18.93	0.15	79	**19**	18.98	0.17	50	**19**	19.07	0.15	38
**h^2^_I_**	**0.5**	0.50	0.06	1411	**0.2**	0.20	0.05	489	**0.1**	0.11	0.04	177
**h^2^_S_**	**0.5**	0.52	0.07	433	**0.2**	0.22	0.06	297	**0.1**	0.15	0.06	110
**h^2^_T_**	**0.5**	0.47	0.11	61	**0.2**	0.33	0.12	48	**0.1**	0.31	0.10	55
***ρ*_g, I-S_**	**0.7**	0.68	0.07	330	**0.7**	0.68	0.16	103	**0.7**	0.67	0.21	76
***ρ*_g, I-T_**	**-0.7**	-0.68	0.12	51	**-0.7**	-0.56	0.24	29	**-0.7**	-0.44	0.31	33
***ρ*_g, S-T_**	**-0.9**	-0.88	0.06	48	**-0.9**	-0.72	0.15	61	**-0.9**	-0.72	0.18	54
***ρ*_p, I-S_**	**0.7**	0.74	0.05	245	**0.7**	0.69	0.04	218	**0.7**	0.72	0.03	212
***ρ*_p, I-T_**	**-0.7**	-0.64	0.13	48	**-0.7**	-0.72	0.09	39	**-0.7**	-0.79	0.08	30
***ρ*_p, S-T_**	**-0.9**	-0.87	0.07	65	**-0.9**	-0.92	0.05	57	**-0.9**	-0.92	0.04	59
***σ* ^2^_e_**	**10**	9.99	0.10	4018	**10**	9.97	0.10	5860	**10**	9.95	0.10	6458

^a ^Marginal Posterior Mean, ^b ^Marginal Posterior standard deviation, ^c ^Effective sample size

**Table 2:** Parameter estimates for 6 parameter scenarios when 10 records were considered per animal (averages over 10 replications)

	**1**				**2**				**3**			
	True	PM^a^	PSD^b^	ESS^c^	True	PM	PSD	ESS	True	PM	PSD	ESS

***μ*_T_**	**19**	19.04	0.20	54	**19**	19.08	0.15	181	**19**	19.15	0.15	174
**h^2^_I_**	**0.5**	0.51	0.04	1683	**0.2**	0.19	0.04	675	**0.1**	0.11	0.03	272
**h^2^_S_**	**0.5**	0.55	0.07	188	**0.2**	0.26	0.07	286	**0.1**	0.11	0.05	79
**h^2^_T_**	**0.5**	0.61	0.17	36	**0.2**	0.43	0.17	58	**0.1**	0.38	0.15	60
***ρ*_g, I-S_**	**0.3**	0.26	0.09	270	**0.3**	0.29	0.18	310	**0.3**	0.37	0.32	51
***ρ*_g, I-T_**	**-0.2**	-0.04	0.21	68	**-0.2**	-0.03	0.34	57	**-0.2**	-0.03	0.43	28
***ρ*_g, S-T_**	**-0.2**	-0.30	0.18	77	**-0.2**	-0.34	0.25	82	**-0.2**	-0.51	0.28	57
***ρ*_p, I-S_**	**0.3**	0.38	0.08	264	**0.3**	0.30	0.05	526	**0.3**	0.29	0.05	289
***ρ*_p, I-T_**	**-0.2**	-0.38	0.26	37	**-0.2**	-0.31	0.18	122	**-0.2**	-0.31	0.15	120
***ρ*_p, S-T_**	**-0.2**	0.00	0.27	51	**-0.2**	-0.17	0.15	139	**-0.2**	-0.15	0.11	175
***σ* ^2^_e_**	**10**	10.07	0.11	3124	**10**	9.94	0.11	7308	**10**	9.99	0.11	6390

	**4**				**5**				**6**			

	True	PM	PSD	ESS	True	PM	PSD	ESS	True	PM	PSD	ESS

***μ*_T_**	**19**	18.98	0.22	28	**19**	19.10	0.25	17	**19**	19.12	0.53	8
**h^2^_I_**	**0.5**	0.49	0.04	795	**0.2**	0.22	0.04	337	**0.1**	0.10	0.03	136
**h^2^_S_**	**0.5**	0.54	0.06	141	**0.2**	0.22	0.05	82	**0.1**	0.12	0.05	63
**h^2^_T_**	**0.5**	0.52	0.11	25	**0.2**	0.37	0.11	26	**0.1**	0.36	0.14	9
***ρ*_g, I-S_**	**0.7**	0.67	0.08	109	**0.7**	0.67	0.11	55	**0.7**	0.70	0.19	23
***ρ*_g, I-T_**	**-0.7**	-0.63	0.13	17	**-0.7**	-0.39	0.25	16	**-0.7**	-0.18	0.31	20
***ρ*_g, S-T_**	**-0.9**	-0.85	0.09	12	**-0.9**	-0.70	0.16	20	**-0.9**	-0.65	0.20	25
***ρ*_p, I-S_**	**0.7**	0.74	0.05	113	**0.7**	0.72	0.03	72	**0.7**	0.72	0.03	57
***ρ*_p, I-T_**	**-0.7**	-0.74	0.12	19	**-0.7**	-0.82	0.08	14	**-0.7**	-0.82	0.07	12
***ρ*_p, S-T_**	**-0.9**	-0.89	0.07	31	**-0.9**	-0.91	0.05	25	**-0.9**	-0.95	0.03	14
***σ* ^2^_e_**	**10**	10.02	0.11	1949	**10**	9.95	0.11	2623	**10**	10.01	0.11	1624

^a ^Marginal Posterior Mean, ^b ^Marginal Posterior standard deviation, ^c ^Effective sample size

As expected, inference efficiency, measured through the marginal posterior standard deviation averages across parameters in Tables 1 and 2 (except residual variance), was reduced as the correlations between underlying variables was reduced. On the contrary, algorithm efficiency, measured through the ESS averages across parameters in Tables 1 and 2 (except residual variance), decreased as correlations increased. In both correlation scenarios, increasing heritability increases inference efficiency for genetic correlations but reduces efficiency for the estimation of heritabilities and environmental correlations. In general, the algorithm average efficiency increases with heritability but some exceptions can be found, particularly under data structure S10.

**Figure 1:**
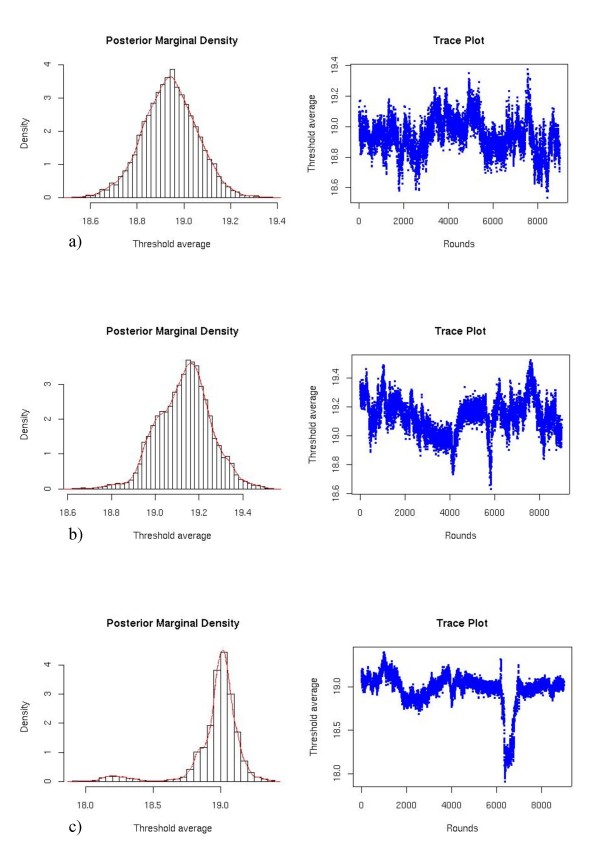
**Marginal posterior distribution and trace plots for the overall mean of the threshold level in three different scenarios for S10**. a) high correlation and high heritability, b) high correlation and medium heritability, c) high correlation and low heritability.

Figure 1 shows the marginal posterior distributions and trace plots for the overall mean of the threshold level obtained in one replication in the scenarios of high correlation and low, medium and high heritabilities when the data structure was S10. The reduction in quality of the chain as heritability decreases can be observed in Tables 1 and 2.

**Figure 2:**
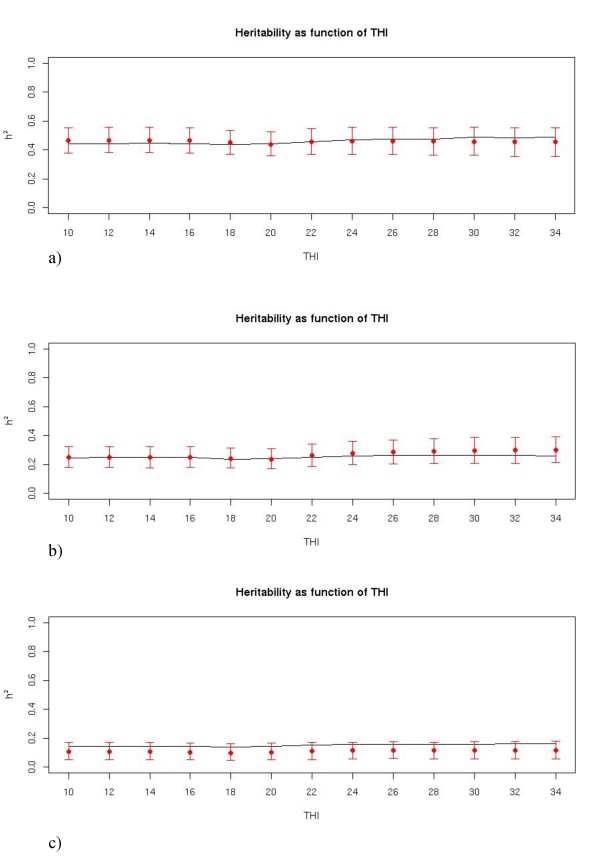
**Patterns of heritability with change in the THI in three different scenarios for S10**. high correlation and high heritability, b) high correlation and medium heritability, c) high correlation and low heritability; the line represents the true pattern, points are the estimated value for the particular THI pattern and the segments represent 95% highest density regions.

Patterns of heritability with change in the THI during the measure day are shown in Figure 2; these plots are estimated from one replication in the scenarios of high correlations and all the cases of heritability with the S10 data structure. Relatively flat patterns were observed, and the 95%HPD region always well covering the true pattern, computed using the approximate formulas as previously described.

Table 3 shows averages across replications of Pearson correlations between predicted and true breeding values for the underlying variables for the 12 investigated scenarios. The predictors were assumed to be the average of the marginal posterior distributions. The observed values of these correlations, *i.e*. accuracies, correspond well with the heritabilities and correlations used during the simulation.

**Table 3:** Pearson correlations between predicted and true breeding in the 12 investigated scenarios (average across replications)

	Number of records per animal = 20					
	1^a^	2	3	4	5	6

Intercept	0.79	0.57	0.78	0.57	0.47	0.44
Slope	0.71	0.51	0.71	0.50	0.43	0.37
Threshold	0.35	0.25	0.65	0.37	0.17	0.26

	Number of records per animal = 10					

	1	2	3	4	5	6

Intercept	0.77	0.59	0.77	0.58	0.46	0.44
Slope	0.63	0.47	0.66	0.47	0.32	0.36
Threshold	0.24	0.16	0.57	0.34	0.12	0.17

^a ^Scenario numbers correspond to headers in Table 1 and 2 where true parameter values can be found

Table 4 shows averages across replications of Pearson correlations between true and predicted values for the underlying random variables defining the model in the first stage of hierarchy, under the 12 investigated scenarios.

**Table 4:** Pearson correlation between predicted and true underlying variables in the 12 investigated scenario (average across replications)

	Number of records per animal = 20					
	1^a^	2	3	4	5	6

Intercept	1.00	1.00	1.00	1.00	1.00	1.00
Slope	0.85	0.84	0.89	0.87	0.84	0.87
Threshold	0.42	0.42	0.81	0.80	0.41	0.80

	Number of records per animal = 10					

	1	2	3	4	5	6

Intercept	0.99	0.99	0.99	0.99	0.99	0.99
Slope	0.75	0.74	0.82	0.82	0.73	0.82
Threshold	0.30	0.31	0.75	0.74	0.30	0.74

^a ^Scenario numbers correspond to headers in Tables 1 and 2 where true parameter values can be found

## Discussion

The model presented in this study provides greater flexibility over traditional reaction norm models when the environmental variable is known, as it allows a semi-parametric form for the reaction norm function. This is a semi-parametric model in the sense that the point in which the linear change is assumed to start is defined by the data themselves. The forms of the functions before and after this point are defined parametrically *a priori*, *i.e*., constant before the change point and a linear function afterwards. To increase flexibility, higher order polynomials or spline functions could be fitted within each one of these two separate periods, with the advantage that within each one of the periods, the functions would remain linear on the parameters. The presented inferential procedure gave unbiased estimates because the uncertain regions always covered the true value of the parameters.

Several alternative algorithms have been proposed for non-parametric or semi-parametric curve fitting. One of them is a Reversible Jump MCMC algorithm where the optimal number of change points (parameters in the model) is estimated [16]. The model presented in this study is a simplified version of this semi-parametric procedure, as the number of parameters is fixed *a priori*. However, the indicated study focuses on fitting averages along the independent variable trajectory; in our case we fit individual sources of variation throughout this trajectory. For this purpose and from a computational point of view, the proposed hierarchical structure is particularly suitable, since the dimension of the problem became greater than when fitting changes in the mean. By using this hierarchical structure, updating mixed model equations in each round of the Gibbs Sampler can be avoided; only the right hand side needs to be modified. In addition, this hierarchical structure jointly with the Bayesian estimation procedure allows for a more appropriate prior assumption that takes advantage of the family structure in the population. Other general procedures for finding change points in continuous functions are the so-called change point techniques. These approaches were previously used in animal breeding to find points of change when fitting heterogeneous residual variance analysing test day milk records [17]. These approaches provide greater flexibility than the models presented because they allow for non-linear functions within each one of the defined regions. However these techniques are more complex because of the non-linearity and the values of two successive functions at change points need to be constrained explicitly to be identical. Our parametrization model can be considered a truncated power representation of a linear spline [18], and in these cases the aforementioned constraints are implicitly considered [19].

Like other previously proposed reaction norm models [2,7,3], the described model could be used for studies and evaluations for genetic tolerance to high heat. The model allows the identification of not only those individuals in the population that are less sensitive to temperature changes after a particular threshold, but also those that became heat stressed at higher values of temperature or THI value. And this individual variation can be partitioned into environmental and genetic components, both for the threshold and the intensity of sensitivity to heat stress. This makes it possible to identify genetically superior individuals for a particular underlying variable of interest: intercept, slope, threshold, or some index involving these variables.

The load function used in this study is the same used for fitting the effect of instantaneous THI on milk production [2]. However it is relatively straight forward to consider more complex functions, for example, those used for studying cumulative effect of THI on carcass weight in pigs [7,3].

In the described model, the covariate (THI) is assumed to be known; however, a traditional reaction norm model could be fitted by predicting an unobserved environmental covariate from the contemporary groups. This extension can be implemented either in two steps as in Kolmodin *et al*. [4] or more complexly as in Su *et al*. [5] by integrating out all the possible values of the contemporary group effects. In these models with unknown covariates, it could be equally reasonable to assume that no effect is observed on the phenotypic performance until some threshold in the environmental scale is reached, beyond which some kind of change in the performance could be expected.

The presented model was applied to study variability on the onset of heat stress tolerance on milk production in dairy cattle. In this study the population size was around 90,000 animals and over 300,000 test-day records were considered. For this data set 250,000 Gibbs iterations took approximately 5.0 CPU days.

Although the methodology presented has been illustrated by focusing on the genetics of heat stress tolerance, more applications could be considered. In particular those longitudinal traits showing a threshold response, *i.e*., those traits with an abrupt change in the response beyond some point on the explanatory variable scale could be fitted using the model presented.

## Conclusion

A model for fitting traits in which the response to an environmental variable is subject to an abrupt linear change was presented. The described statistical procedure performed satisfactorily under the simulated scenarios in estimating the model parameters. As an application example, the model could be useful for identifying animals with higher adaptation to environmental changes, to heat in particular. These animals will be characterized by a smaller phenotypic decline in the performance as well as a later onset of environmental stress. In addition, the proposed methodology can attribute the individual variation on these two expressions of tolerance to environmental stress to genetic and systematic components, which would be useful for the detection of genetically superior breeding animals to be used in selection.

## Competing interests

The authors declare that they have no competing interests.

## Authors' contributions

JPS developed the statistical model, carried out the software implementation, made the simulation design and drafted the manuscript. IM helped with discussion both in theoretical developments and software implementations, as well as in drafting the manuscript. RR contributed with discussion on theoretical aspects and drafting the manuscript.
